# Brief exposure to pulsed magnetic fields supports dental pulp tissue engineering: in vitro evaluation and in vivo proof of concept

**DOI:** 10.1016/j.bbiosy.2026.100151

**Published:** 2026-10-19

**Authors:** Ritika Malhotra, Deby Fajar Mardhian, Laurent Miguel Jia An Lim, Clarice Ferreira Sabino, Baiqing Yu, Nisarat Ruangsawasdi, Pedro Henrique Santaliestra e Silva, Jan Nikolas Iversen, Rafhanah Banu Binte Abdul Razar, Yu Fan Sim, Yee Kit Tai, Ren Jie Jacob Chew, Joao N. Ferreira, Alfredo Franco-Obregón, Vinicius Rosa

**Affiliations:** a Faculty of Dentistry, National University of Singapore, 9 Lower Kent Ridge Road, Singapore, 119085, Singapore; b Department of Dental Materials and Technology, Faculty of Dentistry, Padjadjaran University, Jl. Sekeloa Selatan No. 1, Bandung, Indonesia; c Department of Pharmacology, Faculty of Dentistry, Mahidol University, 6 Yothi Road, Ratchathewi, Bangkok, 10400, Thailand; d Center of Technological Development (CDTec), Federal University of Pelotas, Rua Gomes Carneiro, 01, Pelotas, RS, 96010-601, Brazil; e Department of Surgery, Yong Loo Lin School of Medicine, National University of Singapore, NUHS Tower Block, 1E Kent Ridge Road, Singapore, 119228, Singapore; f Institute of Health Technology and Innovation (iHealthtech), National University of Singapore, MD6, 14 Medical Drive, Singapore, 117599, Singapore; g Biolonic Currents Electromagnetic Pulsing Systems (BICEPS) Lab, National University of Singapore, Singapore, 117599, Singapore; h Center of Excellence and Innovation for Oral Health and Healthy Longevity, Faculty of Dentistry, Chulalongkorn University, 34 Henri-Dunant Road, Wangmai, Pathumwan, Bangkok, 10330, Thailand; i ORCHIDS: Oral Care Health Innovations and Designs Singapore, National University of Singapore, Singapore

**Keywords:** Pulp biology, Biophysics, Odontoblast(s), Oxidative stress, Scaffolds, Bioengineering

## Abstract

Dental pulp tissue engineering requires preservation of dental pulp stem cell (DPSCs) function under adverse conditions while supporting differentiation and tissue formation. Pulsed electromagnetic fields (PEMFs) are non-invasive modulators of cellular function, but their effects on DPSCs cytoprotection under oxidative challenge and on odontoblastic differentiation and pulp-like tissue formation remain unclear. Here, direct PEMF exposure preserved metabolic activity and cytoskeletal organization and reduced intracellular reactive oxygen species (ROS) under moderate oxidative stress while PEMF-conditioned medium preserved membrane integrity, and both accelerated wound closure. PEMF exposure increased intracellular calcium above a time-dependent rise common to exposed and unexposed cells, and this increase was no longer discernible after inhibition of transient receptor potential canonical (TRPC) channels 1, 4 and 5, supporting a TRPC-associated response to the field. PEMF-conditioned medium increased odontogenic marker expression and mineralization in vitro, while transcriptomic analysis identified early changes involving mitochondrial and biosynthetic processes. In a cell-seeded tooth-slice model, cells preconditioned with PEMF-conditioned medium supported the formation of a pulp-like tissue with a collagenous matrix and showed higher vessel density than controls (no PEMF). Collectively, these findings show that PEMF supports DPSCs resilience under oxidative challenge and regenerative functions relevant to pulp tissue formation across complementary experimental models, with potential implications for dental pulp tissue engineering.

## 1 Introduction

Cell-based dental pulp tissue engineering relies on transplanted cells maintaining their function after implantation while supporting differentiation and tissue formation. During early tissue establishment, before a functional vascular supply is established, transplanted cells encounter adverse metabolic conditions that can compromise their function and persistence [1,2]. Cellular resilience to such conditions is therefore relevant to successful tissue formation. Oxidative stress represents one component of this cellular challenge and can impair dental pulp stem cell (DPSC) function through lipid peroxidation, DNA damage, and mitochondrial dysfunction [3,4]. Although pulp cells possess intrinsic antioxidant systems, sustained oxidative stress can overwhelm these defenses. Antioxidant molecules and drugs can mitigate oxidative damage, but their activity depends on local availability and stability [5]. These limitations provide a rationale for non-invasive, non-contact approaches capable of modulating dental pulp cell responses while preserving the regenerative functions required for dental pulp tissue engineering.

Pulsed electromagnetic fields (PEMFs) use bursts of electromagnetic energy with adjustable frequency, intensity, and waveform to modulate cellular function [6,7]. Brief exposures (≤10 min) to low-amplitude (≤3 mT) PEMFs applied in the low frequency range at 15 Hz have been associated with modulation of oxidative stress, mitochondrial adaptation, and cellular resilience, in a response described as magnetic mitohormesis [[8], [9], [10]]. PEMF exposure has also been associated with cellular differentiation and mineralization, while PEMF-induced secretomes from somatic and stem cells can support tissue differentiation and regeneration [8,11,12]. In DPSCs specifically, a single 10 min exposure at 2 mT has previously been shown to enhance neurogenic differentiation, with the response linked to transient receptor potential canonical 1 (TRPC1) channel availability [6]. These findings establish DPSC responsiveness to brief PEMF exposure, but the relevance of this response to oxidative cytoprotection and odontogenic regenerative outcomes relevant to dental pulp tissue engineering remains unknown.

Therefore, the objective of this study was to investigate the effects of PEMF on DPSC cytoprotection under oxidative challenge and on odontoblastic differentiation and pulp-like tissue formation. We hypothesized that brief, low-amplitude PEMF exposure, applied directly or through PEMF-conditioned medium (PEMF CM), protects DPSCs under oxidative challenge and supports their odontoblastic differentiation and pulp-like tissue formation. Our results show that PEMF exerts cytoprotective effects under oxidative challenge and supports odontoblastic differentiation and pulp-like tissue formation, while the acute calcium response and its sensitivity to TRPC inhibition suggest that cells respond directly to the field.

## 2 Materials and methods

### 2.1 Cell culture and ethics

DPSCs (AlCells, California, USA) were cultured using basal growth media (DMEM GlutaMAX and 10% fetal bovine serum (FBS) from Gibco, USA) at 37°C and 5% CO_2_. Cells in passages 4–7 were used for the experiments and passaged at 70–80% confluency. The use of human cells in this study was approved by NUS Institutional Review Board (NUS-IRB-2021-685, 2 November 2021).

### 2.2 Cell exposure to PEMF and production of conditioned media using PEMF

The PEMFs generated were spatially homogeneous, time-varying magnetic fields, consisting of barrages of 20×150 μs on and off pulses, repeated every 6 ms at a frequency of 15 Hz [13,14]. All PEMF exposures lasted 10 min, regardless of field amplitude.

For direct PEMF exposure (*PEMF*), DPSCs were exposed to a field amplitude of 2 mT in basal growth media [6]. For the conditioned-medium experiments (indirect exposure), the basal growth medium of donor DPSCs at 70–80% confluency was replaced with serum-free DMEM GlutaMAX immediately before exposure to 2 mT PEMF for 10 min, and the cells were returned to the incubator for 24 h. The medium was then collected, centrifuged at 1200 rpm for 5 min, supplemented with 10% FBS, and stored at 4°C. This medium was designated *PEMF CM*. To generate the corresponding conditioned-medium control, donor DPSCs were handled identically and placed in the PEMF device for 10 min with the field switched off (0 mT). The medium collected from these cells after 24 h was designated *DPSC CM*. The final FBS concentration was 10% in all groups, the same as in basal growth medium, including the odontogenic cultures, in which odontogenic medium (OdM) and conditioned medium were combined 50:50.

To evaluate the cytoprotective effects of PEMFs, DPSCs in basal growth media were seeded in 96-well plates (5×10^3^ per well) and left undisturbed for 24 h. Thereafter, cells were exposed to tert-butyl hydroperoxide (TBHP, Merck, USA) at concentrations ranging from 100 to 200 µM (IC₅₀ = 152.3 µM, Fig. S2). Different scenarios were tested (Fig. 1A–D):

a. *Pre-PEMF*: to assess whether prior PEMF exposure enhances resilience to oxidative stress, DPSCs were exposed to PEMF 24 h before TBHP challenge.
b. *Pre-PEMF CM*: to determine whether PEMF CM provides protective factors before oxidative stress, DPSCs were treated with PEMF CM for 24 h before TBHP challenge.
c. *Co-PEMF*: to evaluate the immediate protective effects of PEMF during oxidative stress, DPSCs were exposed to PEMF concurrently with TBHP.
d. *Co-PEMF CM*: to test whether PEMF CM modifies cellular responses during oxidative stress, DPSCs were treated with PEMF CM concurrently with TBHP.

**Fig. 1:**
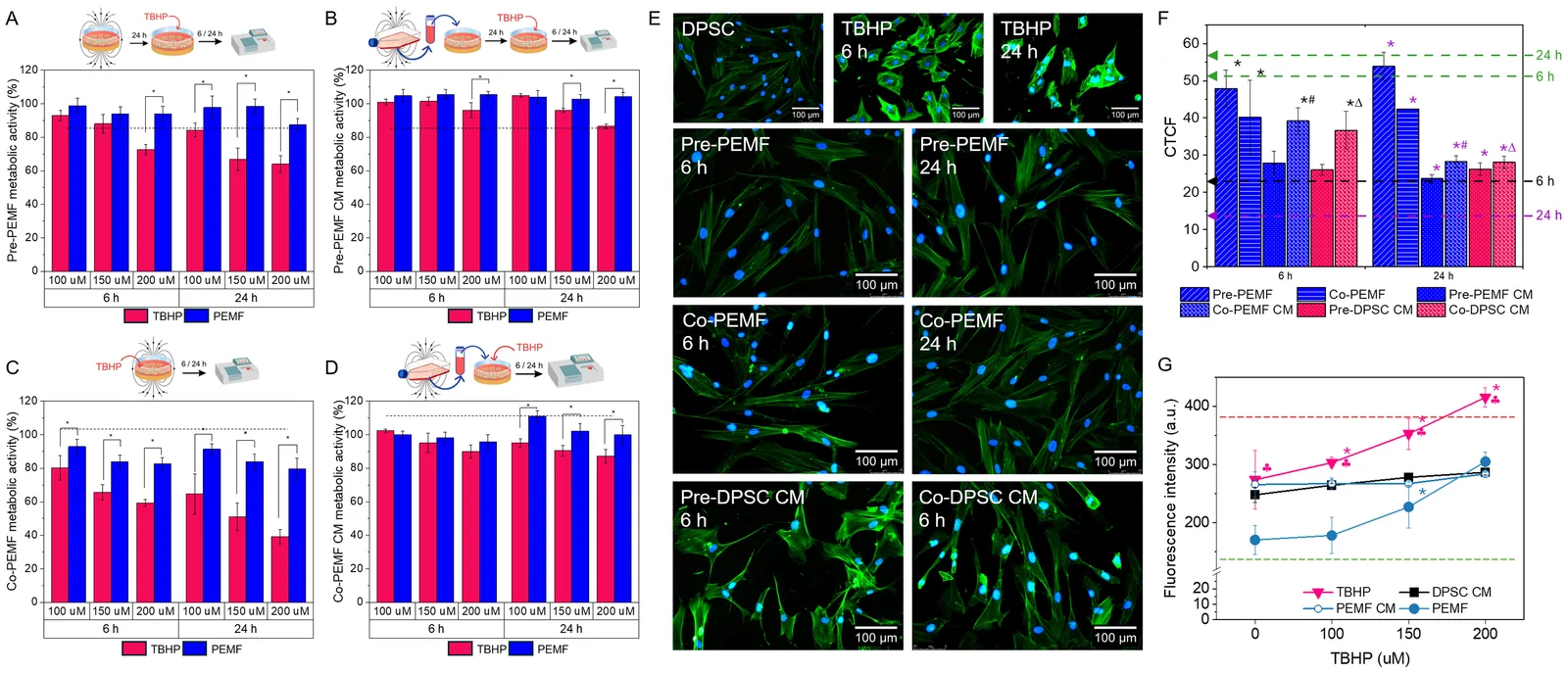
Pulsed electromagnetic fields preserve DPSC metabolic activity and cell shape under an oxidative challenge. (A–D) Metabolic activity (MTS) of DPSCs challenged with tert-butyl hydroperoxide (TBHP; 100, 150 and 200 μM) for 6 or 24 h under the four exposure schedules shown in the schematics: (A) *Pre-PEMF*, (B) *Pre-PEMF CM*, (C) *Co-PEMF*, (D) *Co-PEMF CM*. Red bars, TBHP alone; blue bars, TBHP plus the indicated treatment; values are relative to cells not treated with TBHP (dashed line, 100%). (E) DPSCs stained with phalloidin (F-actin, green) and NucBlue (nuclei, blue) 6 and 24 h after challenge with 150 μM TBHP; six of the eight conditions are shown, and all eight, at larger size, in Fig. S3. (F) Phalloidin fluorescence per cell (corrected total cell fluorescence, CTCF) for the eight conditions; *DPSC CM* is the paracrine control (secretome without the field); CTCF reports fluorescence intensity, not actin content. Dashed lines mark untreated DPSCs and TBHP alone at each time point. (G) Intracellular reactive oxygen species 1 h after challenge, across TBHP concentrations, for TBHP alone, *DPSC CM, PEMF CM* and *PEMF*. Data are mean ± SD, n = 3; two-way ANOVA with Tukey's test (A–D, G) and Dunnett's test (F); * p < 0.05.

Metabolic activity was measured 6 or 24 h after treatment using MTS (Promega, USA, 1:10 ratio, 490 nm, Multiskan FC, Thermo Fisher Scientific, USA). The negative control was DPSCs in basal growth medium. To determine whether scavenging of mitochondrial reactive oxygen species (ROS) reproduces the PEMF-associated response, Mito-TEMPO (10 or 25 µM) was added at the onset of the 150 µM TBHP challenge, and metabolic activity was read after 6 h using WST-1. Results are expressed as fraction of TBHP-induced metabolic-activity loss restored (TBHP-treated group = 0%, untreated control = 100%). Experiments in Fig. 1A–D and in the Mito-TEMPO assay were run as separate experimental series that differed in readout (MTS added without washing in Fig. 1A–D; WST-1 added after one wash in the Mito-TEMPO assay) and in the severity of the injury produced by the same nominal challenge; absolute values are therefore not comparable between the two (Supplementary Material). To evaluate metabolic activity over time, DPSCs were plated (5×10^3^ cells, 96-well plates) in basal growth media, left for 24 h, cultured for up to 21 d in OdM (Fig. S1) combined 50:50 with conditioned medium prepared at 0, 1, 2 or 3 mT, and assessed using WST-1 (Abcam, 1 h incubation at 37°C).

To examine the effects of PEMFs on odontoblastic differentiation and mineralization potential, we treated DPSCs with OdM produced with Biodentine (Septodont, France) discs (10 mm diameter, 1 mm height, set for 24 h at 37°C, 95% humidity) immersed individually in 10 mL of basal growth media for 2 days (37°C, 95% humidity). Afterward, the disc was removed, and the eluate was filtered (0.22 μm, Merck). The OdM was combined in a 50:50 ratio with conditioned medium prepared at 0, 1, 2 or 3 mT, the 0 mT medium being that collected from donor cells placed in the device with the field switched off. The OdM was not supplemented with dexamethasone, β-glycerophosphate or ascorbic acid. The odontogenic stimulus was provided solely by the calcium silicate eluate to drive odontoblastic differentiation and mineralization in DPSCs [15].

To assess cell morphology and cytoskeletal organization, DPSCs were challenged with 150 µM TBHP and analyzed at 6 and 24 h. Direct PEMF exposure was applied either 24 h before TBHP challenge (*Pre-PEMF*) or at the same time as TBHP (*Co-PEMF*). *PEMF CM* was likewise applied either 24 h before TBHP (*Pre-PEMF CM*) or concurrently with TBHP (*Co-PEMF CM*). *DPSC CM* was included as the conditioned-medium control and was applied either 24 h before TBHP (*Pre-DPSC CM*) or concurrently with TBHP (*Co-DPSC CM*). Cells were then stained with phalloidin (ActinGreen™ 488, Invitrogen, USA) for F-actin and with NucBlue™ Live (Invitrogen, USA) for nuclei, and corrected total cell fluorescence (CTCF; Supplementary Material) was calculated.

DPSCs (7×10^3^ cells) were seeded in a 96-well black/clear bottom plate (Thermo Fisher Scientific, USA) and left undisturbed for 24 h. After that, the cells were washed with 1× wash buffer and incubated for 30 min at room temperature. ROS/superoxide detection solution containing TBHP at concentrations of 0, 100, 150, and 200 µM was added to the cells. Alternatively, cells were treated with direct *PEMF* exposure, *PEMF CM* or *DPSC CM* immediately after adding TBHP to the culture, before ROS quantification. ROS were detected using the ROS/Superoxide Detection Assay Kit (Abcam, UK) according to the manufacturer’s instructions. Negative and positive controls for ROS generation and reactions were N-acetyl-L-cysteine (inhibitor) and pyocyanin (inducer), respectively, in basal growth media. Cells were incubated for 1 h in the dark before measuring fluorescence using a microplate reader (Tecan Group Ltd., Switzerland) at 490/525 nm for excitation/emission.

For the wound-closure assay, 70 µL of 5×10^5^ cells/mL was seeded into 3-well culture inserts (Ibidi, Germany) and left undisturbed for 24 h. After that, the inserts were removed, the cells were washed and treated with:

a. *PEMF*: direct exposure to 2 mT PEMF.
b. *PEMF CM*: treatment with conditioned medium collected from donor DPSCs exposed to 2 mT PEMF (indirect exposure).
c. *DPSC CM*: treatment with conditioned medium collected from donor DPSCs not exposed to PEMF, serving as the conditioned-medium control (indirect exposure).
d. Control: fresh basal growth medium.

The assay was followed for up to 120 h and the cell-free area quantified using ImageJ (NIH, USA). As PEMF-induced stem cell differentiation has been shown to occur within amplitude-dependent efficacy windows [11,14], *PEMF CM* generated at 0–3 mT for 10 min was evaluated specifically to identify the most responsive amplitude for odontoblastic differentiation. The relative gene expression of osteocalcin (*OCN*), dentin matrix protein 1 (*DMP-1*), dentin sialophosphoprotein (*DSPP*), matrix extracellular phosphoglycoprotein (*MEPE*), alkaline phosphatase (*ALP*), and collagen type I (*COL-1*) was measured by qRT-PCR using the ∆∆CT method [16]. Mineralization was evaluated by alizarin red S staining (ARS), normalized to Ca deposition per DNA (Supplementary Material). A 50:50 mixture of basal growth media and OdM was the Control (Fig. S1). Single-cell RNA sequencing was performed to characterize early transcriptional responses to a single PEMF exposure at 2 mT (Supplementary Material). DPSCs were harvested after 24 h and processed for single-cell analyses according to standard dissociation and library preparation workflows. Single-cell libraries were sequenced to obtain gene expression profiles at single-cell resolution, enabling comparative transcriptomic analysis between Control and PEMF-treated conditions. The control was DPSCs not exposed to PEMF. *PEMF CM* was selected for the differentiation studies because it produced the fastest wound closure (Section 3). This indirect approach was also better suited to the 21-day differentiation protocol, as repeated direct PEMF exposure would require recurrent removal of cultures from controlled incubation, introducing repeated changes in temperature, CO₂/pH, and handling that could independently affect cell differentiation.

To determine whether DPSCs respond to the field, calcium was measured with Calcium Green-1 AM. Baseline fluorescence was acquired for 2 min 30 s, plates were exposed to PEMF (2 mT, 10 min) or left unexposed, and fluorescence was recorded for another 10 min at 30 s intervals, each value normalized to the well baseline. Recording was repeated after pre-treatment with the TRPC1/4/5 inhibitor Pico145 (10 nM) and its vehicle alone (0.1% DMSO, Supplementary Material). The recordings are reported as group means at each time point.

The membrane integrity, pharmacological rescue, mitochondrial superoxide and catalase assays used the same 150 µM TBHP challenge and compared untreated cells in basal growth medium (Control), TBHP alone, direct PEMF exposure 24 h before TBHP (*Pre-PEMF*) and *PEMF CM* applied for the 24 h preceding TBHP and maintained during the challenge *(PEMF CM*); Mito-TEMPO was included only in the rescue assay. Membrane integrity was assessed by lactate dehydrogenase (LDH) released into culture supernatant 6 h after 150 µM TBHP challenge. A total-lysis control defined the releasable enzyme, and cell-free medium served as the blank (Supplementary Material).

To test whether the field lowers mitochondrial superoxide, this species was measured 1 h after challenge with MitoSOX Red and NucBlue, imaged by confocal microscopy, and expressed as MitoSOX Red intensity per cell (Supplementary Material).

To test whether the response is accompanied by induction of an antioxidant enzyme, catalase activity was measured 6 h after the same challenge in cell lysates against an H₂O₂ standard curve, and normalized to the total protein of the same lysate (Supplementary Material).

### 2.3 *In vivo* pulp-like tissue formation (proof of concept using the tooth-slice model)

The use of human tissues and animals in this research was approved by the NUS Institutional Review Board (NUS-IRB-2021-685, 2 November 2021) and the NUS Institutional Animal Care and Use Committee (R20-0489(A1)24, 23 November 2022) and followed ARRIVE guidelines. For the *in vivo* experiment, 1-mm-thick tooth slices were prepared from eight extracted non-carious human molars obtained from the National University Centre for Oral Health Tooth Tissue Bank, with one slice obtained per tooth. Teeth were collected with the informed consent of the donors, in accordance with the guidelines of the institutional biobank.

Extracted non-carious human molars were mounted on a precision sectioning cutter (Isomet Low Speed Saw, Buehler, USA), and parallel transverse cuts were made at the cervical region under cooling to obtain 1.0-mm-thick slices. Slices were disinfected with 70% (w/v) ethanol for 5 min and stored in distilled water. A 5% (w/v) poly(lactic-co-glycolic acid) solution (PLGA; molecular weight 85,000–160,000, Sigma-Aldrich) was prepared in chloroform overnight. Pulp cavities were filled with NaCl particles (250–425 µm), and the PLGA solution was applied to the cavity. Constructs were maintained for 24 h in a dry cabinet to allow solvent evaporation. The porogen was then removed by four consecutive 24-h immersions in 10 mL distilled water.

Each slice contained a PLGA scaffold and was seeded with 6×10⁶ DPSCs in 100 µL DMEM. Constructs were incubated for 1 h in basal growth medium and then cultured for 48 h in OdM combined 50:50 with either *DPSC CM* (Control) or *PEMF CM* (2 mT), which produced the strongest in vitro differentiation response. Constructs were implanted into dorsal subcutaneous pockets of 5- to 7-week-old female SCID mice. Four mice were used, each receiving one Control and one *PEMF CM* construct in contralateral pockets (n = 4 constructs per group). After 28 d, specimens were retrieved, fixed in 4% formaldehyde, demineralized in 10% formic acid, and serially sectioned for hematoxylin and eosin (H&E), Masson’s Trichrome and Picrosirius Red staining. Sections suitable for analysis were obtained from three constructs per group for H&E and from two constructs per group for Masson’s Trichrome and Picrosirius Red. Vessel density and lumen area were quantified from 10× H&E images in Fiji using one image per construct (n = 3 per group). The tissue region of interest (ROI) was traced from the dentin wall inward, following the tightest boundary enclosing the outermost vascular profiles of the adjacent pulp-like tissue. Individual vascular lumens were traced within the ROI. Vessel density was calculated as vascular profiles per tissue area (vessels/mm²), and lumen area was measured for each vessel.

### 2.4 Statistical analysis

Biological replicates were independent experiments with DPSCs from a single commercial lot. Unless stated otherwise, each assay was run as three independent experiments (n = 3), each in technical triplicate wells, and the mean of the technical replicates of each experiment was the unit of analysis. The exceptions were as follows: the pharmacological rescue assay with Mito-TEMPO was run once with six replicate wells per group, and the wells were the unit of analysis; mitochondrial superoxide was imaged in three fields per replicate and compared on replicate means (Supplementary Material); intracellular calcium was recorded in four independent sets, with the mean of each set as the unit; single-cell RNA sequencing was performed with one library per condition, so its statistics are computed across cells and are exploratory; and in vivo, the construct was the unit of analysis (Section 2.3). We assessed normality using the Shapiro–Wilk test. We compared ROS, metabolic activity, and wound closure by two-way ANOVA with Tukey's test, and CTCF values by Dunnett's test. Gene expression and mineralized matrix were compared by one-way ANOVA with Tukey's test within each time point. For the membrane integrity, pharmacological rescue, mitochondrial superoxide and catalase assays, we used one-way ANOVA with Holm–Sidak's test, using the TBHP-treated group as the reference; lysis and cell-free medium wells were calibration references and were excluded. Vessel density was compared by an unpaired two-sided t-test on image-level values, and lumen area by an exact two-sided Mann–Whitney test on image-level medians. Significance was set at p ≤ 0.05 (SPSS 30.0, IBM, USA; Prism 10.3, GraphPad Software, USA).

## 3 Results

After 6 or 24 h of TBHP exposure, PEMF-treated groups showed higher metabolic activity than the corresponding TBHP-treated groups in most conditions, particularly at 24 h and with concurrent direct exposure (Fig. 1A–D). There was no increase in metabolic activity in cells treated with *PEMF CM* at varying magnetic field strengths compared to the control (0 mT, Fig. S5).

The effect of PEMFs on cytoskeletal organization in DPSCs under oxidative stress was investigated by analyzing cell morphology. Representative images are shown in Fig. 1E and all groups can be found in Fig. S3. Control DPSCs exhibited a spindle shape whereas cells exposed to TBHP showed a disrupted rounded shape. *Pre-PEMF* preserved CTCF at levels comparable to untreated cells at 6 and 24 h, whereas *Co-PEMF* remained intermediate between TBHP and control (p<0.05 vs. both, Fig. 1F). Direct PEMF exposure reduced ROS generation (Fig. 1G, p<0.05). *PEMF CM* also lowered ROS relative to TBHP alone, but *DPSC CM* did so to a similar extent (Fig. 1G).

The wound-closure assay (Fig. 2A–C) showed that complete closure was reached at 72 h with *PEMF CM*, and at 120 h with *PEMF* and with *DPSC CM*, whereas the control reached approximately 85% at 120 h (Fig. 2A shows every group at every time point). *PEMF CM* also showed the highest normalized closure speed (V*_norm_*, Fig. 2D). The ARS (Fig. 2E) showed the highest mineralization for *PEMF CM* at 2 mT at both time points (p < 0.05, Fig. 2F); DPSCs kept in basal growth media showed no mineralization (Fig. S4).

**Fig. 2:**
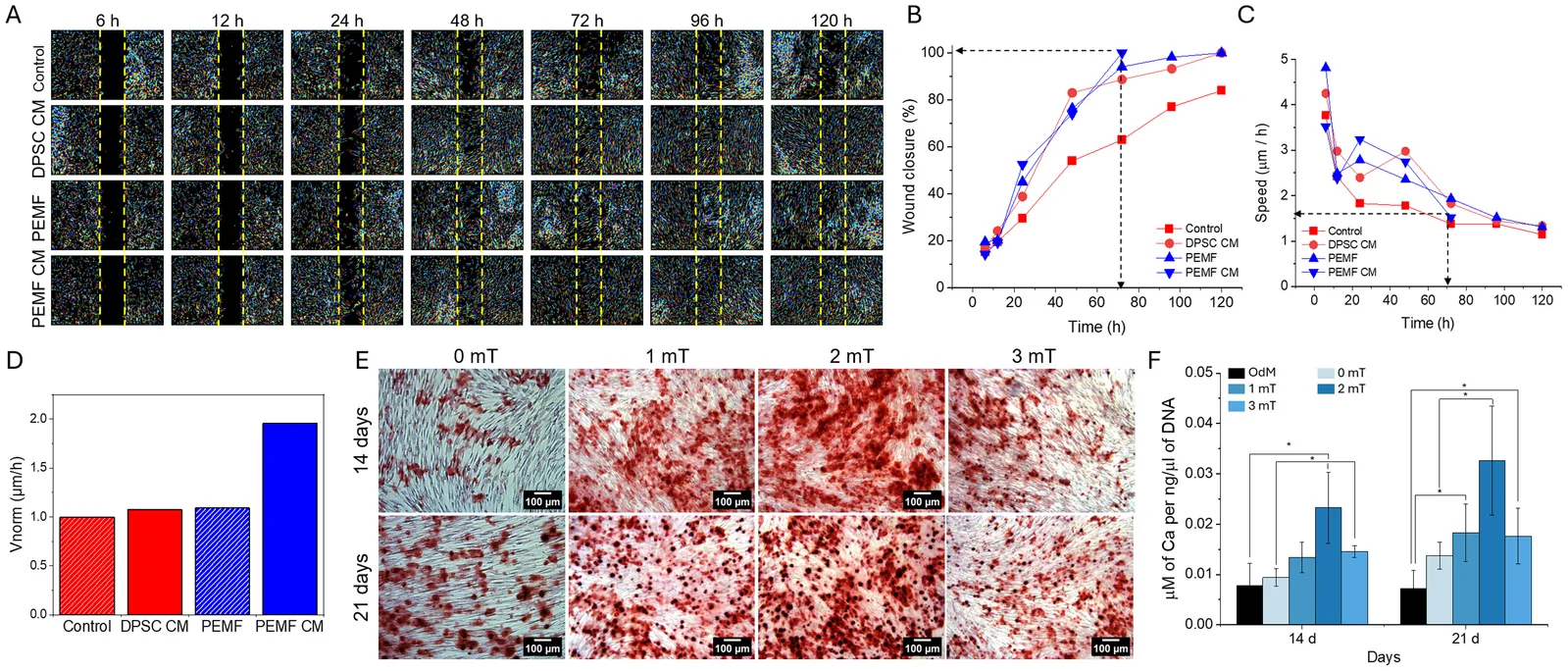
PEMF-conditioned medium accelerates wound closure and supports mineralization. (A) Wound-closure fields from 6 to 120 h for untreated cells in fresh basal medium (Control), *DPSC CM, PEMF* and *PEMF CM*; dashed lines mark the initial cell-free area. (B) Wound closure and (C) closure speed over time for the same groups; *PEMF CM* achieved complete closure at 72 h. (D) Normalized closure speed (V*_norm_*) relative to the Control. (E) Representative alizarin red staining after 14 and 21 d in odontogenic medium (OdM) combined 50:50 with medium conditioned at 0, 1, 2 or 3 mT; scale bars, 100 μm. (F) Mineralized matrix (calcium normalized to DNA content) for the same conditions and for the OdM control (OdM mixed 50:50 with basal growth medium). The micrographs are unnormalized fields of view, whereas the quantification is normalized to the DNA content of each well. Data are mean ± SD, n = 3; wound closure by two-way ANOVA with Tukey's test and mineralized matrix by one-way ANOVA with Tukey's test within each time point; * p < 0.05.

DPSCs treated with *PEMF CM* exhibited increased expression of odontogenic genes at both 14 and 21 d compared with DPSCs cultured in OdM alone. The highest odontogenic response was observed in cells exposed to a 50:50 combination of OdM and 2 mT *PEMF CM* (Fig. 3A).

**Fig. 3:**
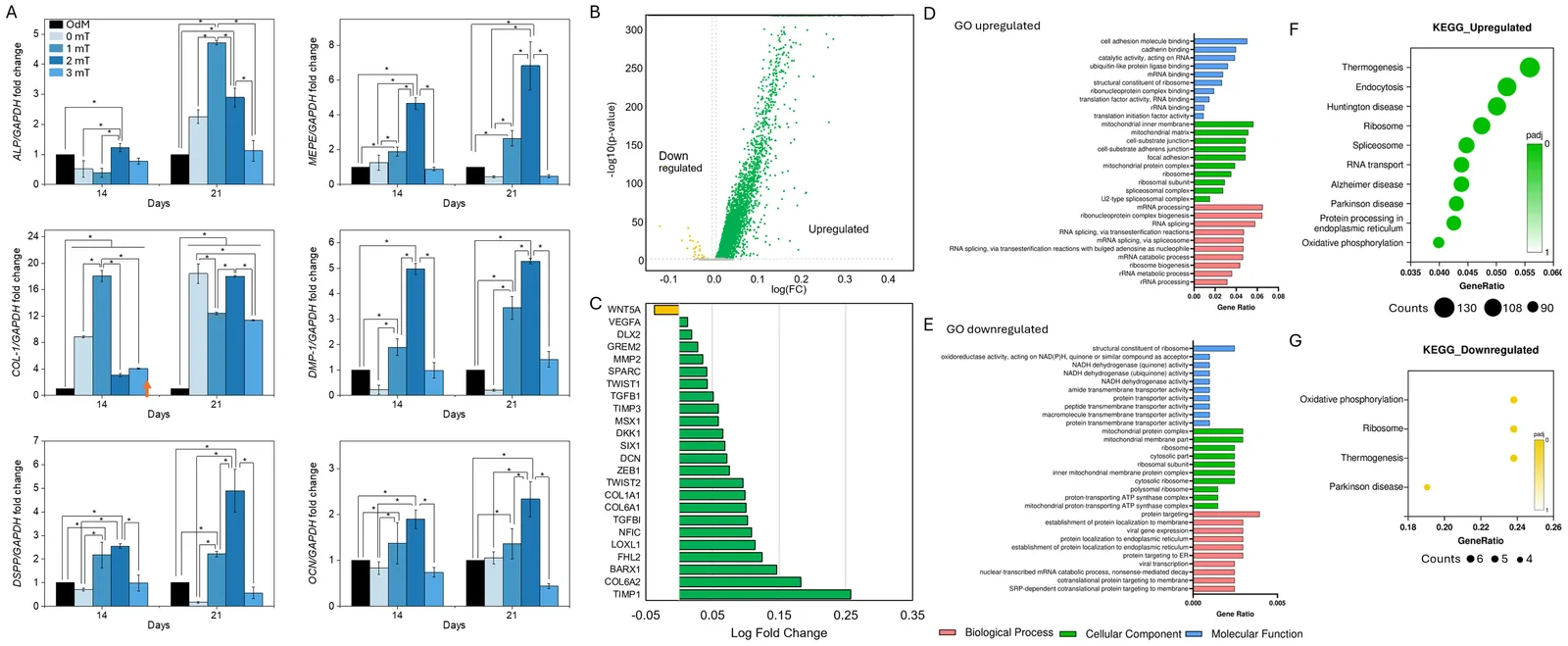
Pulsed electromagnetic fields modulate odontogenic gene expression and transcriptional programs in dental pulp stem cells. (A) Quantitative RT-PCR analysis of markers of odontoblastic differentiation in DPSCs cultured in odontogenic medium (OdM) combined with PEMF-conditioned media (*PEMF CM*) generated at different magnetic intensities (0–3 mT) at 14 and 21 days. Gene expression is shown as fold change relative to OdM alone (control), normalized to GAPDH. Statistical significance is indicated by * at p<0.05. (B) Volcano plot illustrating differential gene expression 24 h after PEMF exposure compared with Control. Genes that are not significantly affected are shown in black (p ≥ 0.05); significantly upregulated genes are shown in green and downregulated genes in yellow. (C) Bar plot of a selection of significantly upregulated (green) and downregulated (yellow) differentially expressed genes (DEGs) relevant to odontogenesis, expressed as log₂ fold change relative to unexposed Control at 24 h following PEMF exposure. (D, E) Gene Ontology enrichment of the upregulated (D) and downregulated (E) DEGs, showing the ten most enriched terms in each of the three ontologies: biological process (pink), cellular component (green) and molecular function (blue), expressed as gene ratio. (F, G) Bubble plot of the enriched Kyoto Encyclopedia of Genes and Genomes (KEGG) pathways among the upregulated (F) and downregulated (G) DEGs. Bubble area is proportional to the number of genes assigned to the pathway and color encodes the adjusted p value. Note the difference in scale between the two panels: the downregulated pathways are supported by four to six genes each, against 90 to 130 for the upregulated ones.

Kyoto Encyclopedia of Genes and Genomes (KEGG) and Gene Ontology enrichment of the differentially expressed genes (DEGs) identified 24 h after exposure indicated enrichment of terms related to protein synthesis and mitochondrial energy metabolism, with ribosome, spliceosome, RNA transport, protein processing in the endoplasmic reticulum and oxidative phosphorylation among the enriched terms (Fig. 3D, 3F; Tables S4 and S5). The corresponding terms among the downregulated genes were each supported by four to six genes, against 90 to 130 for the upregulated pathways (Fig. 3E, 3G).

PEMF-exposed DPSCs exhibited an elevated intracellular calcium signal relative to unexposed controls, superimposed on a gradual time-dependent increase observed in both groups (Fig. 4A). Following pretreatment with the TRPC1/4/5 inhibitor Pico145 (10 nM), the calcium response profiles of PEMF-exposed and unexposed cells largely overlapped (Fig. 4B), indicating that the PEMF-associated increase was no longer clearly discernible, while the time-dependent component common to both groups remained. Vehicle treatment did not prevent the PEMF-associated increase (Fig. S6).

**Fig. 4:**
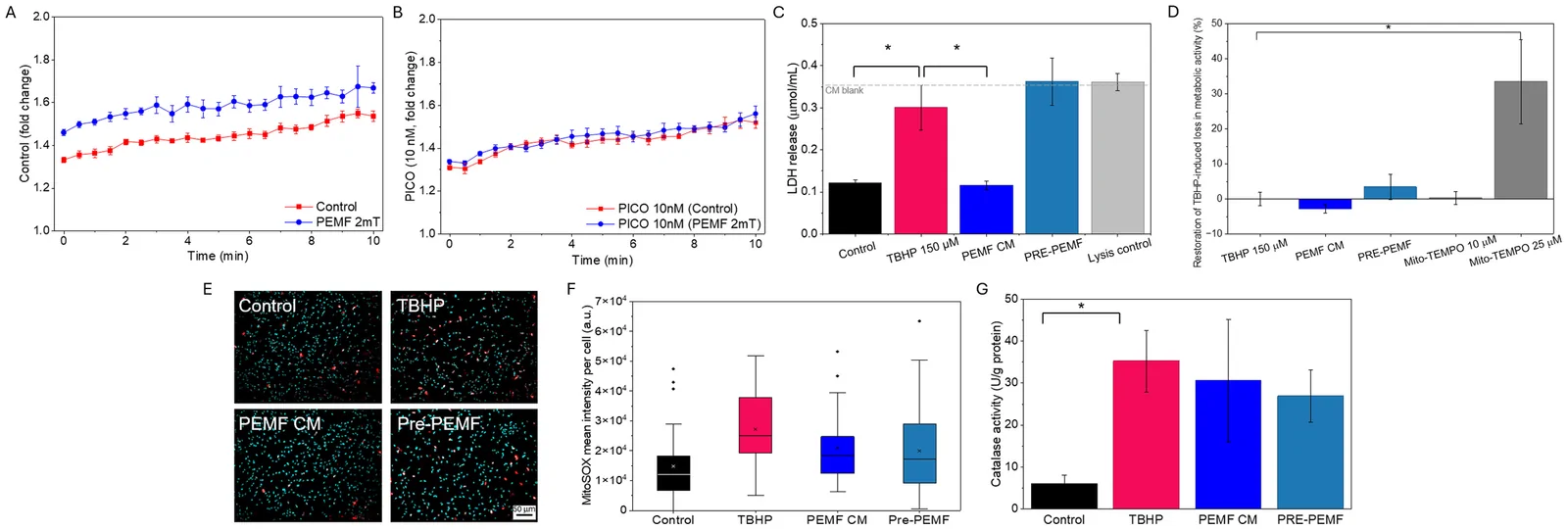
Transduction of the field and boundaries of the cytoprotective response. (A) Intracellular calcium (Calcium Green-1 AM, normalized to the pre-exposure baseline) recorded for 10 min immediately after PEMF exposure (2 mT, 15 Hz, 10 min) or after an equivalent period without exposure. (B) The same recording after a 15 min pre-treatment with the TRPC1/4/5 inhibitor Pico145 (10 nM); the vehicle control is shown in Fig. S6. Data are mean ± SD of four independent sets. (C) Lactate dehydrogenase (LDH) released into the supernatant 6 h after challenge with 150 μM TBHP; all groups except Control received TBHP. The lysis control defines total releasable LDH; cell-free medium blanks were not subtracted and are reported in the Supplementary Material. (D) Pharmacological rescue control with Mito-TEMPO (10 or 25 μM) under the same challenge, in six replicate wells; recovery is the fraction of the TBHP-induced loss in metabolic activity restored (TBHP = 0%, untreated control = 100%); the axis is truncated at 50% and no data are omitted. (E) MitoSOX Red (red) and NucBlue (cyan) 1 h after TBHP; scale bar, 50 μm. (F) Mitochondrial superoxide as MitoSOX intensity per cell; box plots show the distribution of the sampling domains, and the statistical comparison was made between the three biological replicate means, with no difference detected between conditions. (G) Catalase specific activity 6 h after challenge, normalized to the total protein of the same lysate. Data are mean ± SD, n = 3 unless stated; one-way ANOVA with Holm–Sidak's test against the TBHP-treated group; * p < 0.05.

TBHP increased LDH release relative to untreated cells (*p*<0.05) and cells that had received *PEMF CM* beforehand did not (p<0.05 vs. TBHP, Fig. 4C). Neither PEMF condition recovered the metabolic activity (Fig. 4D), whereas the mitochondria-targeted antioxidant Mito-TEMPO restored about one third of it at 25 μM (p<0.05 vs. TBHP). Under the matching condition in Fig. 1 (pre-exposure, 150 µM, 6 h), neither *Pre-PEMF* nor *Pre-PEMF CM* differed significantly from TBHP alone (Fig. 1A–B). No statistically significant differences in mitochondrial superoxide were detected between conditions (Fig. 4E–F; *p*>0.05). The catalase specific activity, which increased with TBHP (*p*<0.05), was not further changed by either PEMF condition (Fig. 4G).

In the tooth-slice model, DPSCs treated with *PEMF CM* supported the formation of pulp-like connective tissue with a vascular network (Fig. 5A). Qualitative analysis suggested a high density of extracellular matrix in the dental pulp-like tissue formed from cells treated with *PEMF CM* (Fig. 5A). Vessel density was higher in the *PEMF CM* constructs than in the Control (p<0.05; Fig. 5B), whereas the median lumen area was smaller, a difference that did not reach significance (p=0.100; Fig. 5C).

**Fig. 5:**
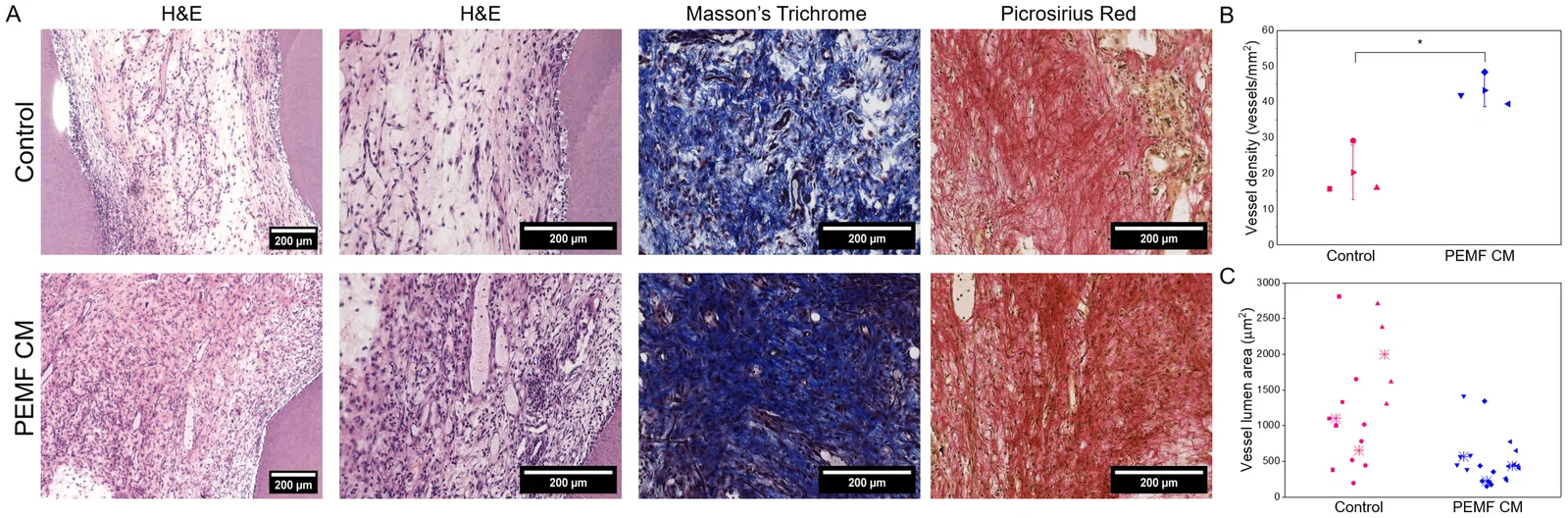
Pulp-like tissue formation and vascularization in the tooth-slice model. (A) Histological sections of constructs retrieved after 28 d, for Control and *PEMF CM*: hematoxylin–eosin at two magnifications, Masson's trichrome, and picrosirius red under bright-field microscopy. Scale bars, 200 μm. (B) Vessel density in the H&E sections; each symbol is one 10× image taken from one slide and the black marker is the group mean ± SD; unpaired two-sided t test, * p ≤ 0.05. (C) Lumen area of the individual vessel profiles in the same images; starred markers are the median of each image, and the comparison was made between the image medians, not between individual vessels, by an exact two-sided Mann–Whitney test, with no significant difference detected.

## 4 Discussion

Dental pulp tissue engineering requires cells that can withstand adverse conditions while retaining their capacity for repair and differentiation. Low-frequency PEMFs have been associated with cellular resilience, metabolic adaptation, and differentiation [10,17]. However, their ability to protect DPSCs under oxidative challenge and, separately, to support odontoblastic differentiation and pulp-like tissue formation remains unknown. Herein, we show that PEMF exerts cytoprotective effects under oxidative challenge and supports regenerative responses relevant to dental pulp tissue engineering.

Under oxidative challenge, direct PEMF exposure before (Fig. 1A) or during TBHP exposure (Fig. 1C) or indirect exposure via *PEMF CM* (Fig. 1B and D) attenuated the loss of metabolic activity relative to TBHP alone, direct exposure reduced intracellular ROS, and pre-exposure also preserved cytoskeletal organization (Fig. 1). Because oxidative injury can compromise plasma membrane integrity, LDH release was used as a marker for membrane damage. *PEMF CM* significantly reduced LDH release (Fig. 4C). The effects of *PEMF CM* may reflect PEMF-induced secretion of cytoprotective factors, as previously reported [18,19]. The *DPSC CM* control distinguished PEMF-associated effects on wound closure (Fig. 2B–D) and odontogenic differentiation and mineralization (Figs. 2F and 3A). However, *DPSC CM* and *PEMF CM* showed comparable effects on ROS and CTCF (Fig. 1F–G), while the metabolic activity and membrane integrity assays lacked a *DPSC CM* control (Figs. 1B, 1D and 4C). PEMF-induced extracellular vesicle release has been reported in myoblasts [19]. However, the potential involvement of extracellular vesicles, cytokines, or growth factors in mediating *PEMF CM* effects in the present study remains speculative, as the presence and types of soluble factors were not investigated.

TBHP disrupts mitochondrial function through ROS accumulation and lipid peroxidation, effects that can be attenuated by mitochondria-targeted antioxidants [20]. We therefore used Mito-TEMPO to test whether mitochondrial antioxidant rescue reproduced the PEMF response. Fig. 4D shows that TBHP-induced injury remained responsive to mitochondrial antioxidant rescue, whereas *Pre-PEMF* did not reproduce this effect. The pronounced reduction in metabolic activity may be partly explained by the washing step. TBHP caused DPSCs to round up (Fig. 1E), and MTS and WST-1 are reduced at the cell surface [21]. In Fig. 1A–D, MTS was added without washing, allowing metabolically active rounded or detached cells to contribute to the measurement. In contrast, washing before WST-1 in Fig. 4D may have removed these cells, leaving approximately 3% of the metabolic activity. Thus, Fig. 4D primarily reflected metabolic activity in cells retained after washing, which was not preserved by *Pre-PEMF*. No significant PEMF-associated modulation of mitochondrial superoxide was detected (Fig. 4E–F). Although catalase provides another defense against peroxide-induced injury [22] and its activity increased in response to TBHP, neither direct PEMF pre-exposure nor *PEMF CM* further enhanced this activity (Fig. 4G).

Together, these findings indicate cytoprotection, reduced intracellular ROS after direct exposure, and preserved cytoskeletal organization after pre-exposure. Magnetic mitohormesis offers one possible explanation, proposing that brief PEMF exposure induces mild mitochondrial stress followed by adaptive responses that increase resistance to injury [[8], [9], [10],^17^]. In myoblasts, PEMF responsiveness has been associated with TRPC1-mediated calcium entry, increased mitochondrial respiration, transient ROS production, and enhanced cellular resilience [10,17]. In DPSCs, PEMF induced a calcium response attenuated by the TRPC1/4/5 inhibitor Pico145, although a time-dependent calcium increase persisted in both groups (Fig. 4A–B). Because Pico145 inhibits TRPC1, TRPC4 and TRPC5, this finding does not establish an exclusive contribution of TRPC1, although previous functional studies in TRPC1-deficient myoblasts support its involvement in PEMF responsiveness [23]. This residual response may involve pathways not modulated by PEMF or nonspecific calcium influx under the assay conditions. Transcriptomic analysis also revealed enrichment of oxidative phosphorylation-related terms among upregulated transcripts (Fig. 3D, 3F). Although these observations are compatible with elements of the mitohormesis model, they do not establish functional mitochondrial adaptation or its contribution to cytoprotection. Moreover, PEMF reduced total intracellular ROS without a significant change in mitochondrial superoxide at the evaluated time point, whereas an early oxidative response has been reported in myoblasts [10,19]. Thus, the calcium and transcriptional responses observed in DPSCs are compatible with elements of the mitohormesis model, whereas the mitochondrial oxidative response differed from that reported in myoblasts under the conditions examined.

The effects observed on wound closure, odontoblastic differentiation, and mineralization are promising for dental pulp tissue engineering. The accelerated wound closure observed in both the *PEMF* and *PEMF CM* groups is consistent with reported PEMF effects on pathways regulating cell migration [[24], [25], [26]], although we do not discard the combined contributions of migration and cell proliferation to the results observed. *PEMF CM* further enhanced odontoblastic differentiation and mineralization when combined with OdM (Fig. 3A). Similar odontogenic responses have been reported with biomaterial-based strategies, including graphene oxide-modified silk fibroin scaffolds [27] and calcium silicate cements such as Biodentine [28]. The gene expression and mineralized matrix presented non-monotonic behavior, increasing from 0 to 2 mT and declining at 3 mT (Figs. 2F and 3A). Although such non-monotonic responses have been discussed in relation to magnetic mitohormesis [17,25,29], the present findings demonstrate an intensity-dependent odontogenic response without establishing a definitive underlying hormetic mechanism. The volcano plot from the transcriptomic analysis 24 h after direct PEMF exposure revealed a predominance of upregulated genes among the identified DEGs (Fig. 3C), including genes associated with extracellular matrix organization, collagen synthesis, mechanotransduction, and mineralization competence (Fig. 3B–C). However, these transcriptional changes do not necessarily reflect functional pathway activation, and protein-level validation was not performed. Furthermore, as transcriptomic analysis was conducted following direct PEMF exposure, whereas differentiation and tissue formation were assessed using *PEMF CM*, any association between these findings remains indirect. The upregulation of NFIC may provide a link with odontogenic differentiation, as NFIC increases ALP activity and collagen I and osteocalcin expression in vitro and functions upstream of canonical dentinogenic markers through regulation of DSPP and DMP1 transcription [30,31]. Increased BARX1 expression, a transcription factor enriched in DPSCs compared with other mesenchymal populations, may reflect pulp-specific developmental programs [32]. Upregulation of COL1A1, COL6A2, SPARC, DCN, and TIMP1 is associated with a collagen-rich and mechanically responsive microenvironment [33,34], while the upregulation of TIMP1 and LOXL1 may indicate matrix preservation and maturation, consistent with their roles in collagen organization and cross-linking [34,35].

DPSCs preconditioned with *PEMF CM* and OdM generated pulp-like tissue *in vivo* (Fig. 5A). Collagen deposition appeared increased with the use of *PEMF CM*, warranting future quantitative assessment of collagen production dynamics (Fig. 5A). Whether the odontogenic and collagen-centric transcriptional changes observed after direct exposure (Fig. 3) relate to the apparent denser collagen fiber deposition in pulp-like tissue formed by DPSCs treated with *PEMF CM* in combination with OdM (Fig. 5A) remains to be tested. There was higher vessel density after treatment with *PEMF CM* (Fig. 5B), with smaller luminal profiles that did not differ significantly (Fig. 5C), consistent with previous reports of increased capillary density and endothelial tubulization in response to PEMF [36,37]. Additional *in vivo* studies with a larger number of animals for higher-fidelity tissue-level characterization are needed to determine whether the tissue formed expresses odontoblastic and dentinogenic matrix markers and to assess blood vessel maturation and collagen deposition.

The present study identified promising PEMF-associated responses relevant to dental pulp tissue engineering, including preservation of DPSC metabolic activity under moderate oxidative injury, reduced intracellular ROS following direct exposure, and enhanced odontogenic differentiation and mineralization. However, the cytoprotective effects were not linked to a specific antioxidant mechanism, and future studies should determine whether they improve cell survival and tissue formation under oxidative or inflammatory conditions in vivo. Transcriptomic analysis also identified changes in genes associated with odontogenic differentiation and extracellular matrix organization. Although transcript abundance does not establish functional pathway activation, single-cell RNA sequencing with one library per condition precludes inference between biological samples. As all experiments used DPSCs from a single commercial lot, the findings may not generalize across donors. Protein-level validation and experiments using DPSCs from multiple donors would help establish the functional relevance and reproducibility of these responses. Finally, the proof-of-concept in vivo study showed pulp-like tissue formation in tooth slices. Further validation in full-length root canal models, with assessment of dentin formation, collagen organization, innervation, and vascular perfusion, would be necessary to determine whether these findings extend to functional regeneration of the pulp–dentin complex.

## 5 Conclusions

Collectively, these findings support the relevance of PEMF as a non-contact modulator of DPSC responses, with effects on cellular resilience and regenerative competence that may help support the biological requirements for dental pulp tissue engineering.

## Ethics declaration

Written informed consent to take part in the study and to publish the article has been obtained from all participants or their legal representatives. The privacy rights of participants have been observed.

This study included organ or tissue donors. This study includes human biological material and consent was obtained by donors, or their next of kin or legal representatives, for use in this study and for publication of the article. The samples used in this research were not sourced from executed prisoners or prisoners of conscience.

This study was performed in compliance with relevant laws, regulatory frameworks and guidelines where the research took place. This study was conducted in accordance with Arrive. This study was approved by the NUS Institutional Review Board . (Approval No. NUS-IRB-2021-685).

This study was conducted in accordance with the ARRIVE (Animal Research: Reporting of In Vivo Experiments) guidelines. This study was approved by the NUS Institutional Animal Care and Use Committee. (Approval No. R20-0489(A1)24).

## Funding

V. Rosa is supported by grants from the Singapore Ministry of Education, Singapore (Academic Research Fund Tier 1, A-8000148-00-00) and the National University Health System, Singapore (NUHSRO/2020/016/RO5+5/Seed-Aug/14). The funders had no role in the study design, data collection, analysis and interpretation, writing of the report, or decision to submit the article for publication.

## Declaration of generative AI and AI-assisted technologies in the manuscript preparation process

During the preparation of this work the authors used ChatGPT (OpenAI, USA) in order to improve the readability of the manuscript. After using this tool/service, the authors reviewed and edited the content as needed and take full responsibility for the content of the published article.

## CRediT authorship contribution statement

**Ritika Malhotra:** Writing – review & editing, Writing – original draft, Methodology, Investigation, Formal analysis, Conceptualization. **Deby Fajar Mardhian:** Writing – review & editing, Writing – original draft, Methodology, Investigation, Formal analysis. **Laurent Miguel Jia An Lim:** Writing – review & editing, Writing – original draft, Methodology, Investigation, Formal analysis. **Clarice Ferreira Sabino:** Writing – review & editing, Writing – original draft, Methodology, Investigation, Formal analysis. **Baiqing Yu:** Writing – review & editing, Writing – original draft, Methodology, Investigation, Formal analysis. **Nisarat Ruangsawasdi:** Writing – review & editing, Writing – original draft, Methodology, Investigation, Formal analysis. **Pedro Henrique Santaliestra e Silva:** Writing – review & editing, Writing – original draft, Methodology, Investigation, Formal analysis. **Jan Nikolas Iversen:** Writing – review & editing, Writing – original draft, Methodology, Investigation, Formal analysis. **Rafhanah Banu Binte Abdul Razar:** Writing – review & editing, Writing – original draft, Methodology, Formal analysis. **Yu Fan Sim:** Writing – review & editing, Methodology, Formal analysis. **Yee Kit Tai:** Writing – review & editing, Methodology, Formal analysis. **Ren Jie Jacob Chew:** Writing – review & editing, Writing – original draft, Methodology, Formal analysis, Data curation. **Joao N. Ferreira:** Writing – original draft, Methodology, Formal analysis. **Alfredo Franco-Obregón:** Writing – review & editing, Supervision, Resources, Methodology, Formal analysis, Conceptualization. **Vinicius Rosa:** Writing – review & editing, Writing – original draft, Supervision, Resources, Project administration, Methodology, Funding acquisition, Formal analysis, Conceptualization.

## Declaration of competing interest

The authors declare the following financial interests/personal relationships which may be considered as potential competing interests: Alfredo Franco-Obregon reports a relationship with QuantumTx Pte. Ltd. that includes: equity or stocks. Alfredo Franco-Obregon has patent System and Method for Applying Pulsed Electromagnetic Fields licensed to QuantumTx Pte. Ltd. The other authors declare that they have no known competing financial interests or personal relationships that could have appeared to influence the work reported in this paper.

## Data Availability

Data will be made available on request.

## Appendix Supplementary materials

Supplementary material: Supplementary material associated with this article is provided in a separate file.
