# Supplementary material for: Brief exposure to pulsed magnetic fields supports dental pulp tissue engineering: in vitro evaluation and in vivo proof of concept
Source: Biomater Biosyst. 2026 Oct 19;24:100151. doi: 10.1016/j.bbiosy.2026.100151 (PMC13756166; doi:10.1016/j.bbiosy.2026.100151)
Supplement: Supplementary file 1 [file mmc1.docx]

Supplementary Material

**Brief Exposure to Pulsed Magnetic Fields Supports Dental Pulp Tissue Engineering: In Vitro Evaluation and In Vivo Proof of Concept**

R. Malhotra, D.F. Mardhian, L.M.L. Jia An, C.F. Sabino, B. Yu, N. Ruangsawasdi, P.H. Santaliestra e Silva, J.N. Iversen, R.B.B.A. Razar, Y.F. Sim, Y.K. Tai, R.J.J. Chew, J.N. Ferreira, A. Franco-Obregón, V. Rosa

**Supplementary methods**

**Dose-response relationship of Tert-butyl Hydroperoxide (TBHP)**

DPSCs were challenged with TBHP (Merck, USA) to ascertain the protective potential of PEMFs against chemically induced oxidative stress. A dose–response assay using TBHP (0–500 µM) was conducted to determine the concentration that reduced metabolic activity by 50% (IC_50_), which was calculated as 152.3 µM. Subsequent experiments were conducted by exposing cells to TBHP at concentrations ranging from 100 to 200 µM.

**Mito-TEMPO, WST-1**

DPSCs (5×10^3^ per well) were seeded in 96-well plates in 100 µL of basal growth media and left undisturbed for 24 h. Mito-TEMPO (10 or 25 µM), a mitochondria-targeted superoxide scavenger, was added at the onset of the 150 µM TBHP challenge. After 6 h the wells were washed once, WST-1 (Abcam) was added and incubated for 2 h at 37 °C, and absorbance was read against cell-free blank wells (n = 6). Recovery is calculated as the fraction of the TBHP-induced metabolic-activity loss that was restored, with the TBHP-treated group set to 0% and the untreated control set to 100%.

**Metabolic activity during odontogenic differentiation**

Metabolic activity was measured by WST-1 at 7, 14 and 21 d in odontogenic medium (OdM) combined with conditioned medium prepared at 0, 1, 2 or 3 mT (n = 3), to establish whether the differentiation response was accompanied by a change in metabolic activity over time (Fig. S5).

**Immunofluorescence**

Cells were fixed with 4% paraformaldehyde, permeabilized with 0.1% Triton-X100, and stained with phalloidin (ActinGreen™ 488, Invitrogen, USA) for F-actin and with NucBlue™ Live (Invitrogen, USA) for nuclei, according to the manufacturer's protocols. DPSCs (1×10⁴ cells) were seeded in 48-well plates and left undisturbed for 24 h before challenge with 150 µM TBHP for 6 or 24 h, alone or with direct PEMF exposure applied before (Pre-PEMF) or during (Co-PEMF) the challenge, with PEMF-conditioned medium on the same schedules (Pre-PEMF CM, Co-PEMF CM), or with medium conditioned by unexposed cells (Pre-DPSC CM, Co-DPSC CM). Control cells were kept in basal growth medium throughout. Fluorescence was quantified per cell as corrected total cell fluorescence, CTCF = ID − (Ac × bg), where ID is the integrated density of the cell, Ac its area and bg the mean fluorescence of a cell-free background region (ImageJ, NIH, USA). The CTCF reports fluorescence intensity and was not calibrated against a known amount of filamentous actin, so it is not reported as actin content. Imaging was performed using an inverted microscope (DMI8, Leica, Germany), and the fluorescence intensity was quantified using ImageJ (NIH, USA). All conditions of a given replicate were stained, imaged and processed in the same session with identical acquisition settings, so that the values support comparison between groups within a replicate.

**Intracellular calcium imaging**

Intracellular calcium was measured with Calcium Green-1 AM (Invitrogen, USA) according to the manufacturer's protocol. DPSCs were seeded at 5 × 10³ cells per well in 96-well black-bottom polystyrene plates (Thermo Fisher Scientific, USA) and maintained in culture until the following day. The culture medium was removed and the cells were loaded with 5 µM Calcium Green-1 AM in FluoroBrite DMEM (Gibco, USA), 100 µL per well, for 1 h in a standard tissue culture incubator, then washed twice with 100 µL FluoroBrite DMEM.

The cells were subsequently incubated for 15 min with 100 µL of FluoroBrite DMEM alone (Control), FluoroBrite DMEM containing 0.1% DMSO (Vehicle Control), or 10 nM Pico145 in FluoroBrite DMEM. Baseline fluorescence was then acquired over 2 min 30 s as five reads 30 s apart, at 506/531 nm excitation/emission on a Cytation 5 microplate reader (BioTek). The plates were then either exposed to PEMF (2 mT, 15 Hz, 10 min) or left unexposed, and fluorescence was measured immediately afterwards for a further 10 min at 30 s intervals. Each value was normalized to the corresponding baseline of the same well, so that the traces report change in intracellular calcium over time rather than absolute concentration.

Pico145 inhibits TRPC1/4/5 channels but it does not isolate TRPC1, and the recordings are therefore reported as evidence that TRPC1/4/5 channels contribute to the response, not that TRPC1 alone carries it; Pico145 attenuated, but did not abolish, the response. The recordings are reported as group means at each time point; no statistical comparison between conditions was performed.

**Membrane integrity (LDH release)**

Culture supernatants were collected 6 h after TBHP addition, before washing, and assayed for lactate dehydrogenase against a standard curve. A total-lysis control was prepared by dissociating untreated DPSCs, centrifuging, resuspending them in culture medium and homogenizing with a Dounce homogenizer to define the total releasable enzyme. Cell-free medium blanks were run in parallel and carried LDH activity (basal medium 0.099 µmol/mL; PEMF-CM 0.351 µmol/mL, the latter derived from the donor cells). Values are reported without subtraction of these blanks. Only the PEMF CM group was maintained in conditioned medium, so its higher medium background acts against, rather than in favor of, the difference reported for that group. The PEMF-CM value is an upper bound on the medium contribution to the PEMF CM wells, because the assayed supernatants had spent 30 h at 37 °C on the cells, over which LDH activity decays.

**Mitochondrial superoxide imaging and quantification**

Mitochondrial superoxide was measured 1 h after TBHP addition with MitoSOX Red and NucBlue. Only the 1 h time point was selected for quantitative analysis because it matched the timing used for the earlier intracellular ROS assessment and captured the mitochondrial superoxide response before more advanced TBHP-induced cellular injury could complicate interpretation of the signal. Multichannel fluorescence hyperstacks were acquired across three independent biological replicates per experimental condition (N = 3), with three fields of view per replicate. Images were processed in ImageJ and the MitoSOX Red and NucBlue channels were split, a uniform global background value was subtracted from the MitoSOX channel, nuclei were counted in the NucBlue channel, and the result was expressed as mean MitoSOX Red intensity per cell in arbitrary units.

Each field was divided into four quadrants to display the full distribution of the signal. Because quadrants within a field, and fields within a replicate, are not independent observations, the statistical comparison was performed on the three biological replicate means rather than on the 36 sampling domains. The result shows the distribution and the test is computed at the level of the experiment.

**Catalase activity**

Six hours after TBHP addition, cells were harvested, homogenized with a Dounce homogenizer and assayed for catalase against an H₂O₂ standard curve. Total protein was determined by BCA in the same lysate, and activity is expressed per gram of protein (n = 3). Because the design compares cells that survived the challenge, it does not distinguish enzyme induction from selection of cells that already had higher catalase activity before the insult. Total protein differed between groups (Control 0.158, TBHP 0.029, PEMF CM 0.034, Pre-PEMF 0.033 mg/mL); the design does not separate enzyme induction from selection of the cells still adherent.

**Alizarin Red quantification**

Cells were washed with phosphate-buffered saline (Invitrogen) and fixed with 4% paraformaldehyde (room temperature, 20 min). After washing with deionized water, cells were stained with 40 mmol/L of alizarin red (Sigma–Aldrich, USA) in distilled water (pH of 4.2 maintained with ammonium hydroxide) and incubated at 37 °C for 30 min. Samples were treated with 10% cetylpyridinium chloride solution (Sigma–Aldrich) at room temperature for 15 min, and the absorbance was measured using a microplate reader (Infinite M200 PRO, Tecan, Germany) at a wavelength of 540 nm. Three individual readings were obtained from each substrate analyzed. The mean absorbance was normalized to genomic DNA content obtained with DNAzol (Invitrogen), and the optical density of the solution was measured using a spectrophotometer (NanoDrop ND-1000 Spectrophotometer, ThermoScientific, USA).

**Single-cell RNA sequencing (scRNA-seq) and transcriptomic analysis**

DPSCs were assigned to two experimental conditions: Control (no PEMF exposure) and PEMF-treated. For the PEMF group, cells were exposed to a single PEMF stimulation (2 mT, 10 min) and subsequently maintained under standard culture conditions for 24 h. After 24 h, cells were harvested and processed for single-cell RNA sequencing (scRNA-seq). Library preparation was performed by Novogene (Singapore) using a droplet-based single-cell workflow (10x Genomics Chromium platform), followed by sequencing on an Illumina instrument. Primary processing, including read alignment to the human reference genome (GRCh38) and generation of gene expression count matrices, was conducted by Novogene using the standard 10x Genomics pipeline (Cell Ranger). Processed count matrices were used for downstream analysis.

Downstream scRNA-seq analyses were performed in R using the Seurat package. Cells and genes were filtered using standard quality-control criteria to remove low-quality barcodes and lowly expressed genes, followed by normalization and scaling. Dimensionality reduction and clustering were performed on the filtered dataset. Differential gene expression analysis was conducted between Control and PEMF-treated groups, and statistically significant genes were defined using adjusted p-values to account for multiple comparisons. Differential expression outputs were visualized as a volcano plot and as log₂ fold-change values for selected genes of interest. To explore the biological functions and pathways associated with PEMF treatment, Gene Ontology (GO) and Kyoto Encyclopedia of Genes and Genomes (KEGG) enrichment analyses were performed for the significantly up- and down-regulated genes, with multiple-testing correction applied to identify significantly enriched terms and pathways. One library was sequenced per condition; enrichment statistics are computed across cells and are presented as exploratory.

**Statistical analysis**

The statistics below apply to the assays reported in Fig. 4C, D, F and G (membrane integrity, pharmacological rescue, mitochondrial superoxide and catalase activity): group differences were assessed by one-way ANOVA followed by Holm-Sidak's multiple comparisons test, with the TBHP-treated group as the reference, since each arm was designed to test whether it alters the response to the challenge. The full output is given in Table S1. All other assays follow the tests stated in the main text.

**Supplementary figures and tables**

Fig. S1. Preparation of odontogenic media (OdM) and mixed proportions with PEMF CM.

Fig. S2. Dose–response of DPSC metabolic activity to tert-butyl hydroperoxide (TBHP). DPSCs were exposed to TBHP at 0–500 µM and metabolic activity was measured after 6 h. The concentration reducing metabolic activity by 50% (IC₅₀) was 152.3 µM; the concentrations used throughout the study (100–200 µM) were selected from this curve.

Fig. S3. Cell morphology and F-actin organization after oxidative challenge. Fluorescence images of DPSCs stained with phalloidin (F-actin, green) and NucBlue (nuclei, blue) after challenge with 150 µM TBHP, for the eight conditions of the assay at 6 and 24 h. Fig. 1E shows representative fields for six of these conditions; no quantification beyond that reported in Fig. 1F is presented. Scale bars, 75 µm.

Fig. S4. DPSCs treated with basal growth media for 21 d do not show signs of mineralization (10×).

Fig. S5. Metabolic activity during odontogenic differentiation. Metabolic activity (WST-1, absorbance at 450 nm) of DPSCs cultured for 7, 14 and 21 d in odontogenic medium combined 50:50 with medium conditioned at 0, 1, 2 or 3 mT. Metabolic activity increased with time in every group and no difference was detected between the conditioned media at any time point.

Fig. S6. Vehicle control for the intracellular calcium recordings. Calcium signal in DPSCs recorded for 10 min immediately after exposure and normalized to the baseline acquired before it, in cells pre-treated for 15 min with the vehicle used to dissolve Pico145 (0.1% DMSO) and either left unexposed (Control) or exposed to PEMF (2 mT). The vehicle did not abolish the field-induced calcium response, indicating that the attenuation of the response observed in the presence of Pico145 (Fig. 4B) is attributable to the inhibitor and not to the solvent.

Table S1. Statistical output for the quantitative panels of Fig. 4. One-way ANOVA followed by Holm-Sidak's multiple comparisons test against the TBHP-treated group.

| **Panel** | **ANOVA** | **Comparison vs TBHP** | **Adjusted p** |
| --- | --- | --- | --- |
| C — LDH | F(3,8) = 30.4; p = 0.0001 | Control | 0.0013 |
|  |  | PEMF CM | 0.0013 |
|  |  | Pre-PEMF | 0.096 |
| D — Metabolic activity | F(5,30) = 224.7; p < 0.0001 | Control | < 0.0001 |
|  |  | PEMF CM | 0.76 |
|  |  | Pre-PEMF | 0.76 |
|  |  | Mito-TEMPO 10 µM | 0.94 |
|  |  | Mito-TEMPO 25 µM | < 0.0001 |
| F — MitoSOX | F(3,8) = 1.89; p = 0.21 | Control | 0.13 |
|  |  | PEMF CM | 0.35 |
|  |  | Pre-PEMF | 0.35 |
| G — Catalase | F(3,8) = 6.44; p = 0.016 | Control | 0.011 |
|  |  | PEMF CM | 0.54 |
|  |  | Pre-PEMF | 0.48 |

Table S2. Individual replicate values underlying Fig. 4 (biological replicates, except the metabolic activity values of Fig. 4D, which are six replicate wells of a single experiment).

| **Assay** | **Group** | **Replicate values** |
| --- | --- | --- |
| LDH release (µmol/mL) | Control | 0.1225 · 0.1273 · 0.1115 |
|  | TBHP | 0.3610 · 0.2839 · 0.2575 |
|  | PEMF CM | 0.1263 · 0.1138 · 0.1056 |
|  | Pre-PEMF | 0.4251 · 0.3416 · 0.3181 |
|  | Lysis control | 0.3828 · 0.3431 · 0.3577 |
| Metabolic activity (% of control) | Control | 82.9 · 96.4 · 100.3 · 108.1 · 105.6 · 106.8 |
|  | TBHP | 1.5 · 3.9 · 5.0 · 2.4 · 1.5 · 5.7 |
|  | PEMF CM | −0.6 · 1.5 · 0.8 · 0.7 · 2.1 · −0.9 |
|  | Pre-PEMF | 13.1 · 5.1 · 5.0 · 3.9 · 8.1 · 4.4 |
|  | Mito-TEMPO 10 µM | 2.6 · 5.4 · 2.1 · 2.1 · 3.2 · 6.4 |
|  | Mito-TEMPO 25 µM | 29.8 · 50.5 · 42.5 · 16.7 · 37.0 · 38.0 |
| MitoSOX per cell (a.u.), replicate means | Control | 12731 · 25010 · 7443 |
|  | TBHP | 29925 · 21961 · 30787 |
|  | PEMF CM | 21462 · 21519 · 21219 |
|  | Pre-PEMF | 27946 · 20179 · 12020 |
| Catalase (U/g protein) | Control | 8.01 · 6.19 · 3.86 |
|  | TBHP | 27.92 · 42.69 · 34.99 |
|  | PEMF CM | 43.46 · 33.52 · 14.77 |
|  | Pre-PEMF | 20.20 · 27.87 · 32.52 |

Table S3. Sensitivity of the comparisons that did not reach significance. The minimum detectable difference is the smallest difference the design could have resolved with 80% power at α = 0.05, given the pooled standard deviation of the assay.

| **Panel** | **Comparison** | **Observed difference** | **Minimum detectable difference** |
| --- | --- | --- | --- |
| C — LDH (µmol/mL) | Pre-PEMF vs TBHP | +0.061 | 0.103 |
| D — Metabolic activity (%) | PEMF CM vs TBHP | −2.7 | 10.7 |
|  | Pre-PEMF vs TBHP | +3.3 | 10.7 |
|  | Mito-TEMPO 10 µM vs TBHP | +0.3 | 10.7 |
| F — MitoSOX (a.u.) | Control vs TBHP | −12,496 | 16,923 |
|  | PEMF CM vs TBHP | −6,158 | 16,923 |
|  | Pre-PEMF vs TBHP | −7,509 | 16,923 |
| G — Catalase (U/g protein) | PEMF CM vs TBHP | −4.6 | 23.0 |
|  | Pre-PEMF vs TBHP | −8.3 | 23.0 |

For each comparison, the minimum detectable difference was computed for 80% power at α = 0.05 from the pooled standard deviation and the ANOVA degrees of freedom. In all cases it exceeded the observed difference; these results are therefore reported as failures to detect an effect at the achieved sensitivity, not as evidence of equivalence.

Table S4. KEGG pathways enriched among the differentially expressed genes 24 h after PEMF exposure, in the order shown in Fig. 3F and 3G.

| **Direction** | **Pathway** |
| --- | --- |
| Upregulated | Thermogenesis |
|  | Endocytosis |
|  | Huntington disease |
|  | Ribosome |
|  | Spliceosome |
|  | RNA transport |
|  | Alzheimer disease |
|  | Parkinson disease |
|  | Protein processing in endoplasmic reticulum |
|  | Oxidative phosphorylation |
| Downregulated | Oxidative phosphorylation |
|  | Ribosome |
|  | Thermogenesis |
|  | Parkinson disease |

Gene ratio ranged from 0.040 to 0.056 among the upregulated pathways, each supported by 90 to 130 genes, and from 0.19 to 0.25 among the downregulated pathways, each supported by 4 to 6 genes.

Table S5. Gene Ontology terms enriched among the differentially expressed genes 24 h after PEMF exposure, in the order shown in Fig. 3D and 3E. BP, biological process; CC, cellular component; MF, molecular function.

| **Direction** | **Ontology** | **GO ID** | **Term** | **Gene ratio** | **Adjusted p** |
| --- | --- | --- | --- | --- | --- |
| Upregulated | BP | GO:0006364 | rRNA processing | 0.0315 | 1.55e-29 |
| Upregulated | BP | GO:0016072 | rRNA metabolic process | 0.036 | 2.26e-30 |
| Upregulated | BP | GO:0042254 | ribosome biogenesis | 0.0436 | 6.73e-42 |
| Upregulated | BP | GO:0006402 | mRNA catabolic process | 0.0465 | 6.22e-28 |
| Upregulated | BP | GO:0000377 | RNA splicing, via transesterification reactions with bulged adenosine as nucleophile | 0.0469 | 4.19e-39 |
| Upregulated | BP | GO:0000398 | mRNA splicing, via spliceosome | 0.0469 | 4.19e-39 |
| Upregulated | BP | GO:0000375 | RNA splicing, via transesterification reactions | 0.0473 | 2.94e-39 |
| Upregulated | BP | GO:0008380 | RNA splicing | 0.0579 | 7.13e-45 |
| Upregulated | BP | GO:0022613 | ribonucleoprotein complex biogenesis | 0.0647 | 7.55e-55 |
| Upregulated | BP | GO:0006397 | mRNA processing | 0.0651 | 3.25e-45 |
| Upregulated | CC | GO:0005684 | U2-type spliceosomal complex | 0.0151 | 2.74e-22 |
| Upregulated | CC | GO:0005681 | spliceosomal complex | 0.0276 | 1.60e-31 |
| Upregulated | CC | GO:0044391 | ribosomal subunit | 0.0291 | 6.47e-38 |
| Upregulated | CC | GO:0005840 | ribosome | 0.0353 | 5.48e-41 |
| Upregulated | CC | GO:0098798 | mitochondrial protein complex | 0.0389 | 2.66e-42 |
| Upregulated | CC | GO:0005925 | focal adhesion | 0.0488 | 2.96e-29 |
| Upregulated | CC | GO:0005924 | cell-substrate adherens junction | 0.0488 | 4.43e-29 |
| Upregulated | CC | GO:0030055 | cell-substrate junction | 0.049 | 1.86e-28 |
| Upregulated | CC | GO:0005759 | mitochondrial matrix | 0.0515 | 6.31e-26 |
| Upregulated | CC | GO:0005743 | mitochondrial inner membrane | 0.0563 | 3.02e-35 |
| Upregulated | MF | GO:0003743 | translation initiation factor activity | 0.0091 | 2.16e-11 |
| Upregulated | MF | GO:0019843 | rRNA binding | 0.0097 | 6.39e-12 |
| Upregulated | MF | GO:0008135 | translation factor activity, RNA binding | 0.0145 | 6.52e-15 |
| Upregulated | MF | GO:0043021 | ribonucleoprotein complex binding | 0.019 | 4.00e-19 |
| Upregulated | MF | GO:0003735 | structural constituent of ribosome | 0.0265 | 4.32e-33 |
| Upregulated | MF | GO:0003729 | mRNA binding | 0.0275 | 1.11e-12 |
| Upregulated | MF | GO:0044389 | ubiquitin-like protein ligase binding | 0.0321 | 2.16e-11 |
| Upregulated | MF | GO:0140098 | catalytic activity, acting on RNA | 0.0388 | 3.22e-12 |
| Upregulated | MF | GO:0045296 | cadherin binding | 0.0398 | 3.91e-21 |
| Upregulated | MF | GO:0050839 | cell adhesion molecule binding | 0.0505 | 3.02e-15 |
| Downregulated | BP | GO:0045047 | protein targeting to ER | 0.003 | 1.57e-05 |
| Downregulated | BP | GO:0072599 | establishment of protein localization to endoplasmic reticulum | 0.003 | 1.57e-05 |
| Downregulated | BP | GO:0070972 | protein localization to endoplasmic reticulum | 0.003 | 3.17e-05 |
| Downregulated | BP | GO:0006605 | protein targeting | 0.004 | 6.17e-05 |
| Downregulated | BP | GO:0006614 | SRP-dependent cotranslational protein targeting to membrane | 0.0025 | 1.08e-04 |
| Downregulated | BP | GO:0006613 | cotranslational protein targeting to membrane | 0.0025 | 1.08e-04 |
| Downregulated | BP | GO:0019080 | viral gene expression | 0.003 | 1.08e-04 |
| Downregulated | BP | GO:0000184 | nuclear-transcribed mRNA catabolic process, nonsense-mediated decay | 0.0025 | 2.30e-04 |
| Downregulated | BP | GO:0019083 | viral transcription | 0.0025 | 0.0013 |
| Downregulated | BP | GO:0090150 | establishment of protein localization to membrane | 0.003 | 0.0013 |
| Downregulated | CC | GO:0022626 | cytosolic ribosome | 0.0025 | 4.70e-05 |
| Downregulated | CC | GO:0044455 | mitochondrial membrane part | 0.003 | 5.41e-05 |
| Downregulated | CC | GO:0098800 | inner mitochondrial membrane protein complex | 0.0025 | 5.70e-05 |
| Downregulated | CC | GO:0098798 | mitochondrial protein complex | 0.003 | 6.15e-05 |
| Downregulated | CC | GO:0005753 | mitochondrial proton-transporting ATP synthase complex | 0.0015 | 8.96e-05 |
| Downregulated | CC | GO:0045259 | proton-transporting ATP synthase complex | 0.0015 | 8.96e-05 |
| Downregulated | CC | GO:0044391 | ribosomal subunit | 0.0025 | 1.19e-04 |
| Downregulated | CC | GO:0042788 | polysomal ribosome | 0.0015 | 1.94e-04 |
| Downregulated | CC | GO:0044445 | cytosolic part | 0.0025 | 2.60e-04 |
| Downregulated | CC | GO:0005840 | ribosome | 0.0025 | 2.60e-04 |
| Downregulated | MF | GO:0003735 | structural constituent of ribosome | 0.0025 | 4.96e-04 |
| Downregulated | MF | GO:0008320 | protein transmembrane transporter activity | 9.88e-04 | 0.0166 |
| Downregulated | MF | GO:0022884 | macromolecule transmembrane transporter activity | 9.88e-04 | 0.0166 |
| Downregulated | MF | GO:1904680 | peptide transmembrane transporter activity | 9.88e-04 | 0.0248 |
| Downregulated | MF | GO:0008565 | protein transporter activity | 9.88e-04 | 0.0248 |
| Downregulated | MF | GO:0042887 | amide transmembrane transporter activity | 9.88e-04 | 0.0255 |
| Downregulated | MF | GO:0003954 | NADH dehydrogenase activity | 9.88e-04 | 0.0255 |
| Downregulated | MF | GO:0008137 | NADH dehydrogenase (ubiquinone) activity | 9.88e-04 | 0.0255 |
| Downregulated | MF | GO:0050136 | NADH dehydrogenase (quinone) activity | 9.88e-04 | 0.0255 |
| Downregulated | MF | GO:0016655 | oxidoreductase activity, acting on NAD(P)H, quinone or similar compound as acceptor | 9.88e-04 | 0.0379 |

Gene ratio ranged from 0.009 to 0.065 among the upregulated terms and from 0.001 to 0.004 among the downregulated terms.
